# Efficacy and safety of different intensities of anticoagulation with warfarin in elderly patients with non-valvular atrial fibrillation

**DOI:** 10.12669/pjms.42.3.14504

**Published:** 2026-03

**Authors:** Minghong Yan, Shandong Mao, Hongping Zhao, Liping Wang, Shuiquan Wang

**Affiliations:** 1 Minghong Yan Department of Nursing, Chun‘an First People‘s Hospital, Zhejiang Provincial People‘s Hospital Chun‘an Branch, Hangzhou, Zhejiang Province 311700, P.R. China; 2 Shandong Mao Department of Pharmacy, Chun‘an First People‘s Hospital, Zhejiang Provincial People‘s Hospital Chun‘an Branch, Hangzhou, Zhejiang Province 311700, P.R. China; 3 Hongping Zhao Department of Cardiovascular Medicine, Chun‘an First People‘s Hospital, Zhejiang Provincial People‘s Hospital Chun‘an Branch, Hangzhou, Zhejiang Province 311700, P.R. China; 4 Liping Wang Department of Cardiovascular Medicine, Chun‘an First People‘s Hospital, Zhejiang Provincial People‘s Hospital Chun‘an Branch, Hangzhou, Zhejiang Province 311700, P.R. China; 5 Shuiquan Wang Department of Orthopedics, Chun‘an First People‘s Hospital, Zhejiang Provincial People‘s Hospital Chun‘an Branch, Hangzhou, Zhejiang Province 311700, P.R. China

**Keywords:** Anticoagulation, Different Intensities, Elderly, Non-Valvular Atrial Fibrillation, Warfarin

## Abstract

**Objective:**

To evaluate the efficacy and safety of different intensities of warfarin anticoagulant therapy in elderly patients with non-valvular atrial fibrillation (NVAF)

**Methodology:**

This retrospective study was conducted at Chun’an First People’s Hospital. Clinical records of 108 elderly patients with NVAF who received warfarin anticoagulant therapy from January 2022 to August 2023 were included. Among them, 54 patients were assigned to the low-intensity anticoagulation group (international normalized ratio [INR] 1.6-2.0), and 54 patients comprised the standard-intensity anticoagulation group (INR 2.1-3.0). All patients were followed up for 24 months. The creatinine clearance (Ccr) before treatment and at 3, 6, 12, and 24 months after treatment was assessed. Incidents of thromboembolism and bleeding in the two groups during the follow-up period were compared.

**Results:**

There were no statistically significant differences in the Ccr levels between the two groups before treatment and at each time point after treatment (*P*>0.05). No statistically significant differences were observed between the two groups in terms of the incidences of ischemic stroke, systemic embolism, non-fatal myocardial infarction, and all-cause mortality (*P*>0.05). The total incidence of bleeding events in the low-intensity group was lower than that in the standard-intensity group (*P*<0.05).

**Conclusions:**

The results of this study indicate that low-intensity warfarin anticoagulation has similar renal function outcomes (as assessed by creatinine clearance) to standard-intensity anticoagulation, with no increase in the risk of thromboembolism. However, low-intensity anticoagulation is associated with a lower risk of bleeding.

## INTRODUCTION

Atrial fibrillation (AF), one of the most common arrhythmic diseases in clinical practice, mainly occurs in the elderly patients.^1^-^3^ With the gradual aging of the population, the incidence of AF is on the rise.^1^,^4^ The prevalence of AF rises significantly with age, increasing from 0.5% in individuals aged 40 years to 5%-15% in those aged 80 years.^4^ In addition, chronic diseases such as coronary heart disease, heart failure, and hypertension are closely associated with the development of AF.^5^ Non-valvular atrial fibrillation (NVAF) accounts for more than 65% of all atrial fibrillation cases.^2^-^5^ Elderly patients with NVAF are often present with a hypercoagulable state, making them prone to thromboembolism, especially ischemic stroke.^4^,^5^ Therefore, the primary goal of treating elderly NVAF patients is anticoagulation to eliminate thrombi, improve blood stasis, and reduce the risk of mortality.

Currently, commonly used anticoagulant drugs include warfarin, dabigatran, and rivaroxaban.^5^,^6^ Among them, warfarin, a frequently used oral anticoagulant, is characterized by good efficacy and low cost.^7^ It exerts its anticoagulant effect mainly by inhibiting the synthesis of vitamin K-dependent coagulation factors in the liver.^7^ The international normalized ratio (INR) is an important indicator for monitoring the anticoagulant effect of warfarin.^7^,^8^ Guidelines for AF in Europe and North America^9^,^10^ recommend maintaining the INR range between 2.0 and 3.0, as this range minimizes the risk of ischemic stroke and bleeding. However, due to the decline in physical function and metabolic levels in elderly NVAF patients, some studies^11^,^12^ have suggested controlling the INR within a relatively lower range. This study compared outcomes of elderly NVAF patients treated with low-intensity anticoagulation (INR: 1.6-2.0) and standard-intensity anticoagulation (INR: 2.1-3.0), aiming to identify the optimal INR range for this population of AF patients.

## METHODOLOGY

This retrospective study was conducted at the Chun‘an First People‘s Hospital, and included records of 108 elderly patients with NVAF who received warfarin anticoagulant therapy from January 2022 to August 2023. Among them, 54 patients were treated with low-intensity anticoagulation (INR: 1.6-2.0) and 54 were treated with standard intensity anticoagulation (INR: 2.1-3.0).

### Ethical Approval:

The ethics committee of Chun’an County First People’s Hospital approved this retrospective study with the number 2024-04-12-54; Date: December 30, 2024. The ethics committee reviewed the study protocol and specifically assessed data security, patient confidentiality, and integrity of the medical records. Given the retrospective design and the use of de-identified data extracted from hospital records, the requirement for written informed consent was formally waived by the ethics committee.

### Inclusion criteria:

- ≥ 60 years old.
- Meets the diagnostic criteria for coronary heart disease in the “Guidelines for Diagnosis and Treatment of Stable Coronary Heart Disease”.^2^
- Meets the diagnostic criteria for NVAF in the “Primary Guidelines for Cardiac Rehabilitation of Coronary Heart Disease (2020)”.^3^
- CHA2DS 2-VASc score ≥ 2.
- Warfarin is used as an antithrombotic therapy.

### Exclusion criteria:

- Damage to liver and kidney function, with transaminase levels exceeding three times the upper limit of normal reference values, and endogenous creatinine clearance rate (Ccr) less than 30 ml/min.
- Dual antiplatelet therapy.
- Patients with abnormal coagulation function.
- Patients with a history of stroke.
- Patients with a history of gastrointestinal bleeding.
- Patients with a history of malignant tumor diseases.
- Follow up not completed.

### Treatment method:

The initial dose of warfarin (manufacturer: Shanghai Shangyao Xinyi Pharmaceutical Co., Ltd.; specification: 2.5mg per tablet) was 2.5mg/d, and the dose was adjusted according to the patient’s INR value during the medication process. The specific adjustment method was as follows: starting from the third day of medication, INR was measured. If INR <1.5, the dose of warfarin was increased by 0.625mg/d. If INR >3.0, the dose of warfarin was reduced by 0.625mg/d. If INR was within the target range, the original dose was maintained. INR was measured again 3-5 days after adjusting the dosage until stabilized within the target range. After reaching the target range, the test was conducted once a week. If the target range was met twice in a row, the INR measurement was changed to once every four weeks. Both groups were followed up for 24 months.

### Treatment continuity:

Throughout the 24-month follow-up, warfarin therapy was continued under regular INR monitoring. No patient permanently discontinued warfarin. Temporary dose modifications were made in response to INR fluctuations as part of routine anticoagulation management under clinical supervision, without deviation from the assigned INR target range.

### Observation indicators:

- Creatinine clearance (Ccr) level was determined as follows: Fasting blood and 24-hour urine were collected from patients in the morning. The picric acid method was used to detect blood creatinine (Scr) and urine creatinine (Ucr), and Ccr was calculated. Ccr = [(140 − age) × body weight (kg)] / [72 × Scr (mg/dL)] (× 0.85 for females), according to the Cockcroft–Gault formula.^13^ The impact of warfarin anticoagulant therapy on renal function in patients was evaluated by regular monitoring of Ccr.
- The primary endpoint events included ischemic stroke, systemic embolism, non-fatal myocardial infarction, and all-cause mortality.
- The safety endpoint events included upper gastrointestinal bleeding, subcutaneous bruising, cerebral hemorrhage, hematuria, and gingival bleeding.

### Statistical analysis:

SPSS/PC statistical software (version 24.0; IBM Corp, Armonk, NY, USA) was used for all analyses. The count data were presented as n (%), and the differences between groups were analyzed using the chi-square test. Visual (histogram and probability plot) and analytical (Kolmogorov-Smirnov/Shapiro-Wilk test) methods were used to evaluate whether variables follow a normal distribution. The measurement data that conformed to a normal distribution were represented in the form of mean ± standard deviation (SD), and independent sample t-test was used to determine the difference between the two groups. Non-normally distributed data were represented by median and interquartile range (IQR), and the Mann-Whitney U test was used for intergroup comparisons. The statistical significance was set to P<0.05. PRISM 8.0 software (GraphPad, San Diego, USA) was used to draw Ccr variation curves.

## RESULTS

This study analyzed data of 108 elderly patients with NVAF who received warfarin anticoagulant therapy. There were 60 males in the cohort, accounting for 55.6%. The patients were aged 60–89 years, with a median age of 71 years (IQR: 66–79 years). Of 108 patients, 54 received low-intensity anticoagulation (INR: 1.6-2.0) treatment, and 54 received standard intensity anticoagulation (INR: 2.1-3.0) treatment. There was no statistically significant difference in baseline data between the two groups of patients (*P*>0.05) (Table-I).

**Table-I:** Comparison of baseline data between two groups of patients.

Baseline data	Low-intensity group (n=54)	Standard-intensity group (n=54)	χ^2^/ Z/t	P
Male (Yes), n(%)	31 (57.4)	29(53.7)	0.150	0.699
Age (Years), M(IQR)	68 (66-72)	69.5 (65-75)	-0.747	0.456
BMI (kg/m²)	23.1±2.6	23.8±3.6	-1.168	0.246
Hypertension (Yes), n(%)	12 (22.2)	18 (33.3)	1.662	0.197
Diabetes (Yes), n(%)	3 (5.6)	7 (13.0)	1.763	0.184
CHA2DS2-VASc score	3 (3-4)	3 (3-4)	0.949	0.295

***Note:*** BMI, body mass index; χ^2^, Chi-square test; Z, Mann-Whitney U test; t, independent-sample t test.

**Fig.1:**
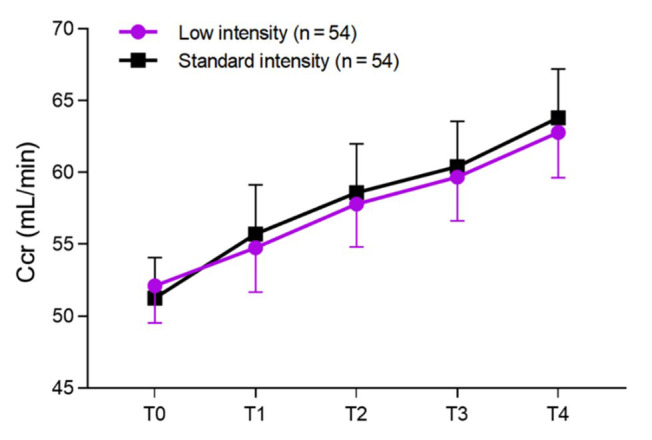
Note: T0, Before treatment; T1, After 3-months of treatment; T2, After 6-months of treatment; T3, After 12-months of treatment; T4, After 24-months of treatment.

As shown in Fig.1, there were no statistically significant differences in Ccr levels between the two groups before treatment and at 3, 6, 12, and 24 months after treatment (P > 0.05). During the 24-month follow-up period, the occurrence of major endpoint events in the two groups was recorded in detail. In the low-intensity group, there were two cases of ischemic stroke, four cases of systemic embolism, two cases of non-fatal myocardial infarction, and one case of all-cause mortality, with an incidence rate of 16.7% (9/54). In the standard-intensity group, there was one case of ischemic stroke, two cases of systemic embolism, one case of non-fatal myocardial infarction, and zero cases of all-cause mortality, with an incidence rate of 7.4% (4/54). Although the number of events in the low-intensity group was higher than that in the standard-intensity group (9 vs. 4), there was no statistically significant difference between the two groups (*P* > 0.05) (Table-II).

**Table-II:** Occurrence of two main endpoint events.

Variables	Low-intensity group (n=54)	Standard-intensity group (n=54)	χ^2^	P
Ischemic stroke	2 (3.7)	1 (1.9)	-	1.000**
Systemic embolism	4 (7.4)	2 (3.7)	-	0.678**
Non-fatal myocardial infarction	2 (3.7)	1 (1.9)	-	1.000**
All-cause mortality	1 (1.9)	0 (0)	-	1.000**
Total number of occurrences	9 (16.7)	4 (7.4)	2.186	0.139*

***Note:***

* Pearson’s Chi-square test;

** Fisher’s Exact Test.

The total number of bleeding events in the low-intensity group was 4, including one case of subcutaneous ecchymosis, one case of cerebral hemorrhage, and two cases of bleeding gums. The total number of bleeding events in the standard-intensity group was 14, including four cases of upper gastrointestinal bleeding, three cases of subcutaneous ecchymosis, two cases of cerebral hemorrhage, two cases of hematuria, and three cases of bleeding gums. The overall incidence of the low-intensity group was significantly lower than that of the standard intensity group (7.4% vs. 25.9%) (*P*<0.05) (Table-III).

**Table-III:** Occurrence of safety endpoint events in two groups.

Variables	Low-intensity group (n=54)	Standard-intensity group (n=54)	χ^2^	P
Upper gastrointestinal bleeding	0 (0)	4 (7.4)	-	0.118**
Subcutaneous ecchymosis	1 (1.9)	3 (5.6)	-	0.618**
Cerebral hemorrhage	1 (1.9)	2 (3.7)	-	1.000**
Hematuria	0 (0)	2 (3.7)	-	0.495**
Bleeding gums	2 (3.7)	3 (5.6)	-	1.000**
Total number of occurrences	4 (7.4)	14 (25.9)	6.667	0.010*

***Note:***

* Pearson’s Chi-square test;

** Fisher’s Exact Test.

## DISCUSSION

This study retrospectively analyzed the outcomes of elderly patients with NVAF treated with low-intensity (INR: 1.6-2.0) and standard-intensity (INR: 2.1-3.0) warfarin anticoagulation. The results showed that the Ccr levels of the two groups were comparable during the 24-month treatment period. This finding indicates that varying intensities of warfarin anticoagulation do not cause significant damage to renal function in elderly NVAF patients. There was no statistically significant difference in the total incidence of thromboembolic events between the two groups. However, the total incidence of bleeding events in the low-intensity group was significantly lower than that in the standard-intensity group.

The results of the study suggest that controlling the INR range within 1.6-2.0 is reasonable for anticoagulant therapy in elderly NVAF patients. This is consistent with the findings of studies by Zhang et al.^12^ and Ye et al.^14^ From a pharmacological perspective, warfarin exerts its anticoagulant effect mainly by inhibiting the synthesis of vitamin K-dependent coagulation factors, and does not act directly on the kidneys. Thus, its impact on renal function is relatively minimal.^7^,^8^,^12^,^14^ The Ccr remained relatively stable in both groups during treatment, which suggests that neither low-intensity nor standard-intensity warfarin anticoagulation regimens cause significant adverse effects on renal function in elderly NVAF patients. It should be noted that renal function in elderly patients may already be impaired to some extent, and their renal reserve and compensatory capacities are relatively weak.^12^,^14^

During the 24-month follow-up, there was no statistically significant difference in the total incidence of thromboembolic events between the two groups. This indicates that in terms of preventing cardiovascular and cerebrovascular diseases, low-intensity warfarin anticoagulation achieved comparable efficacy to standard-intensity therapy, with both regimens reducing the risk of cardiovascular and cerebrovascular events in elderly NVAF patients to a certain extent. This is consistent with previous research findings.^15^,^16^ Warfarin exerts its anticoagulant function by inhibiting the synthesis of coagulation factors, reducing the risk of thrombosis, and thereby decreasing the incidence of cardiovascular and cerebrovascular diseases.^15^-^17^ Although the target INR ranges differed between the two groups, both achieved a certain level of anticoagulant effect, effectively inhibiting thrombosis and thus exhibiting similar effects in preventing cardiovascular and cerebrovascular diseases. It is important to note that multiple factors may impact the occurrence of cardiovascular and cerebrovascular diseases in elderly NVAF patients. Beyond thrombosis, the risk of such events is closely associated with patients’ underlying diseases (such as hypertension, diabetes, and hyperlipidemia), lifestyle factors (such as smoking, alcohol consumption, and lack of exercise), and genetic factors.^16^,^18^,^19^

In this study, the total number of bleeding events in the low-intensity group was significantly lower than that in the standard-intensity group. This indicates that low-intensity warfarin anticoagulation has a certain advantage in reducing the bleeding risk in elderly NVAF patients. It is well-known that a higher anticoagulant intensity is associated with a higher bleeding risk.^20^,^21^ Moreover, elderly patients themselves have poor vascular elasticity, may have abnormalities in coagulation function, and exhibit low tolerance to bleeding.^22^ Therefore, low-intensity warfarin anticoagulation not only ensures a certain anticoagulant effect but also reduces the risk of bleeding, which meets the clinical needs of elderly NVAF patients.

These results have significant implications for clinical practice. For elderly NVAF patients, especially those at high bleeding risk (such as advanced age, comorbidity with multiple underlying diseases, or hepatic/renal insufficiency), low-intensity warfarin anticoagulation should be prioritized. This approach can effectively prevent thromboembolic events while minimizing the bleeding risk, thereby improving treatment safety and patients’ quality of life.

Strengths of this study include the strict stratification of warfarin anticoagulation into predefined low- and standard-intensity INR target ranges, enabling a direct comparison of efficacy and safety in an elderly non-valvular atrial fibrillation population. In addition, the 24-month follow-up provides relatively long-term real-world outcome data. Importantly, we evaluated both renal function (Ccr) and bleeding outcomes, offering a more comprehensive assessment of treatment safety in older adults, a group frequently underrepresented in randomized trials.

Future studies are warranted to validate and extend these findings. In particular, prospective multicenter investigations with larger sample sizes are needed to improve external validity and reduce bias. Incorporating anticoagulation quality indicators such as time in therapeutic range (TTR), the proportion of INR values within target range, and INR variability would further strengthen the interpretation of treatment intensity. Comparative studies against newer oral anticoagulants (NOACs) may also help clarify the optimal anticoagulation strategy for elderly patients with NVAF.

### Limitations:

This single-center retrospective study with a modest sample size is vulnerable to selection and information bias and residual confounding; therefore, findings should be interpreted as associations rather than causal effects. The low number of primary endpoint events may have limited power and increased the risk of type II error, and non-significant results should not be taken as equivalence. Key warfarin-control metrics (time in therapeutic range, INR-in-range proportion, INR variability) and precise event dates were not consistently available, precluding robust anticoagulation-quality assessment and time-to-event analyses. Renal function was assessed using Cockcroft–Gault creatinine clearance, which may reduce comparability with eGFR-based studies. Bleeding was not graded using standardized major/minor criteria because of limited documentation. Multiple outcomes were tested without formal multiplicity adjustment, and other anticoagulants were not evaluated. Larger multicenter prospective studies with standardized definitions and complete longitudinal data are needed.

## CONCLUSION

This retrospective study compared the efficacy and safety of low-intensity (INR 1.6–2.0) versus standard-intensity (INR 2.1–3.0) warfarin anticoagulation in elderly patients with non-valvular atrial fibrillation. Under the conditions of this study, low-intensity anticoagulation was associated with comparable observed thromboembolic outcomes and a lower incidence of bleeding, while renal function (assessed by creatinine clearance) remained stable during follow-up. Given the retrospective single-center design, limited event numbers, and potential residual confounding, these findings should be interpreted cautiously and considered hypothesis-generating. Anticoagulation intensity may be individualized according to each patient’s clinical characteristics and risk profile, and further prospective multicenter studies are warranted.

### Recommendations:

Future multicenter, large-sample studies are needed to assess the optimal INR range for different subtypes of NVAF patients and comprehensively evaluate the reliability and safety of warfarin in elderly NVAF patients.

### Authors’ contributions:

**MY:** Literature search, study design and manuscript writing.

**SM, HZ, LW and SW:** Data collection, data analysis and interpretation. Critical Review

**MY:** Manuscript revision and validation and is responsible for the integrity of the study.

All authors have read and approved the final manuscript.

***Funding:*** Hangzhou Science and Technology Bureau (2024ZDSJ0202).
